# A Moment-of-Inertia-Dependent Surface Homogenization Method for Porous Polymer Beams

**DOI:** 10.3390/polym18080979

**Published:** 2026-04-17

**Authors:** Renqiang Xiang, Shuo Li, Ming Zhang, Li Li

**Affiliations:** State Key Laboratory of Intelligent Manufacturing Equipment and Technology, School of Mechanical Science and Engineering, Huazhong University of Science and Technology, Wuhan 430074, China

**Keywords:** metamaterial beams, surface effect, homogenization method, multiscale method, moment-of-inertia-dependent surface, nonclassical mechanics

## Abstract

Obvious size-dependent bending responses are observed in porous polymer beams, particularly as their thickness approaches the scale of the lattice constant. However, the relationship between the size dependency and the microstructure remains unclear. Direct numerical simulations are computationally expensive due to the complexity of the microstructures, while classical multiscale methods, which neglect the surface effect, yield results that deviate significantly from actual behavior. In this study, an equivalent model for porous polymer beams incorporating surface-driven moment of inertia is developed to capture the size-dependent Young’s modulus by introducing a surface strength factor and surface thickness. Then, an online prediction framework based on the offline dataset generated by the moment-of-inertia-dependent surface homogenization method was established for size-dependent bending response. The proposed framework is evaluated in terms of accuracy and computational efficiency. Results show that the classical multiscale homogenization method can produce relative errors as high as 1108%, whereas the surface homogenization method maintains relative errors below 4%. Moreover, the computational cost is substantially reduced compared to direct numerical simulations. This work not only uncovers the underlying moment-of-inertia-dependent surface mechanism of the size-dependent behavior in metamaterial beams but also delivers an accurate and efficient tool for their structural design and performance prediction.

## 1. Introduction

Metamaterials are artificially engineered materials that exhibit unconventional and often extraordinary properties [1,2,3], including superior mechanical, acoustic, and thermal characteristics [4,5,6,7], and have attracted increasing interest in recent years due to the rapid advancement of fabrication techniques, particularly the widespread adoption of 3D printing. These developments have enabled their application and in-depth investigation in diverse fields such as aerospace, civil engineering, and biomedical systems [8,9,10,11]. Among various structural forms, metamaterial beams, with their tailorable stiffness and programmable mechanical responses, have demonstrated remarkable advantages in intelligent systems for vibration isolation and energy harvesting [12,13], and further exhibit broad applicability in the design of load-bearing components in engineering structures [14,15,16].

Existing studies have demonstrated the presence of size-dependent effects in metamaterials [17,18,19], which is critical for evaluating the deformation behavior of metamaterial components. The size effect becomes pronounced when metamaterial structures are subjected to shear or bending loads [20,21,22], and the fewer the number of lattice units along the thickness direction of a beam structure, the more significant the size dependence observed during bending deformation [23,24]. This behavior deviates from the size-dependent characteristics exhibited under tensile deformation [25,26], as bending stiffness exhibits markedly higher sensitivity to structural dimensions than tensile stiffness [27]. However, when analyzing the deformation of metamaterial beams with complex microstructures, discretizing the beam using a refined mesh and solving with high-fidelity finite element methods requires substantial computational resources [28,29], and may even lead to convergence issues or unsolvable models. Therefore, an efficient and accurate homogenization method for predicting the deformation behavior of metamaterial beams would be highly beneficial.

Homogenization method is a numerical procedure that extracts mechanical properties from a microscale representative volume element that characterizes the overall behavior of the metamaterial. After homogenization, the equivalent mechanical properties can be employed to model and simulate the entire lattice as a homogeneous material at the macroscale, thereby allowing for the generation of simpler meshes, leading to significant reductions in computational time. Originally, homogenization theory was introduced to accelerate simulations of composite materials, and it is now widely applied to predict the effective mechanical behavior of metamaterial structures [30,31,32].

Numerous homogenization methods based on classical mechanics have been developed to date. Among them, the first-order asymptotic expansion method is one example [33], which is founded on the concept of multiscale expansion, expanding the displacement field as a series in slow and fast variables, and obtaining the macroscopic constitutive relations by solving a set of microscopic problems. Another important approach is the variational homogenization method [34,35], which is based on the principle of minimum potential energy, systematically applying variational formulations to multiscale problems. Both methods are studied based on the representative volume element (RVE) framework [36], and when dealing with media possessing periodic microstructures, the RVE must be significantly smaller than the characteristic macroscopic length scale, so that the microscopic and macroscopic behaviors can be regarded as two distinct yet coupled systems throughout the entire structure, the macroscopic variables are approximated as constants at the microscale, thereby enabling separation of variables for solution [37,38]. Additionally, semi-analytical approaches such as self-consistent methods and mean-field theories are frequently employed to determine the effective properties of non-periodic multiphase materials. These methods introduce equivalent media or embedded microstructural models, establishing the connection between microscale and macroscale via statistical averaging principles [39,40]. In practical numerical computations, finite element methods (FEM) based on RVEs have become the most commonly used homogenization tools in engineering applications. The key to this approach lies in the proper definition of boundary value problems for the RVE [41,42,43]. The RVE method employs finite element techniques to model a single periodic unit cell, applying periodic boundary conditions (PBC) to simulate the response behavior of an infinite structure [44,45], thereby extracting the macroscopic equivalent stiffness tensor or constitutive relations [46]. These classical homogenization methods perform well when dealing with periodic structures and mesoscale problems, yet they all assume that material responses are locally continuous, neglecting nonlocal effects and boundary layer influences arising from comparable microstructural and macroscopic length scales. Therefore, when the structural scale approaches or is smaller than the microstructural scale, or when materials exhibit pronounced size-dependent behaviors (such as softening or stiffening during bending), classical methods often fail to accurately predict their responses [47,48].

With the advancement of experimental and simulation studies, numerous studies have demonstrated that when the characteristic size of the microstructure approaches the overall geometric scale of the structure, traditional scale-separation-based homogenization methods fail to accurately predict the mechanical behavior of metamaterials [49,50,51]. To address this, researchers have gradually developed a series of homogenization methods based on non-classical continuum theories, aiming to overcome the limitations of classical models, and more realistically capture nonlocal responses, boundary layer effects, and micro-macro coupling characteristics in metamaterials. The Gurtin-Murdoch surface elasticity theory was the first mathematical framework to describe material mechanical behavior at the microscale, extending classical elasticity theory by introducing independent “surface energy” or “surface stress” on the bulk material surface to characterize mechanical behavior at micro- and nano-scales [52,53]. Strain gradient theory, on the other hand, introduces higher-order derivatives of strain, establishing nonlocal constitutive relations embedded with intrinsic length scale parameters, thus overcoming the limitation of the classical Cauchy model’s “zero characteristic length” [54,55]. Additionally, the micropolar theory established by Mindlin and Tiersten [56] and the strain gradient plasticity theory proposed by Fleck [57] provide important foundations for such models. Homogenization methods based on these theories can more accurately describe size effects manifested when the structural thickness or characteristic length decreases to the scale of the microstructure [58]. Another important class of methods, which is based on Cosserat or micropolar continuum theory, considers that material points possess not only translational degrees of freedom but also independent rotational degrees of freedom, introducing additional couple stresses and rotational stiffness parameters. Such theories are particularly suited to describe structures in which significant local rotations, voids, hinges, or shear lag phenomena occur within the unit cell, such as honeycomb or shear-dominated metamaterials [59,60,61]. Although homogenization theories based on non-classical mechanics can capture size effects and nonlocal responses to some extent, when applied to metamaterials with complex microstructures—such as in studies of how the lattice microstructure and geometry affect the mechanical behavior of 3D-printed polymer materials—the filling geometry (cubic, helical, linear, etc.) and the filling density can significantly influence the structural mechanical performance [62]. In such cases, it is often challenging to accurately determine model parameters, such as internal length scales and higher-order elastic moduli, and the choice of these parameters can have a substantial impact on the predictive accuracy of the models.

At the micro- and nano-scales, surface effects, which may arise from surface stress, surface elasticity, and residual surface stress, have been extensively studied [63,64], whereas at the macroscopic scale, the surface effect in metamaterials results from the interaction between microscopic features and the deformation of the overall structure [65]. In the study of the bending performance of composite materials, a number of theoretical and experimental works have provided valuable insights. For example, investigations on the flexural behavior of functionally graded carbon nanotube reinforced composite sandwich beams have shown that material gradation and structural configuration can significantly influence the bending stiffness and deformation characteristics of the beams [66]. Meanwhile, experimental and analytical studies on carbon nanotube reinforced textile-based composites have also demonstrated that nanoscale reinforcements can effectively enhance the flexural performance and load-bearing capacity of the structures [67]. Moreover, previous studies on the surface effect in metamaterial structures have predominantly focused on macroscopic tensile deformation [68,69], with relatively few investigations addressing the influence of the surface effect on metamaterial beams under macroscopic bending deformation, which has considerably limited the structural design and performance prediction of metamaterial beams in engineering applications. Clearly, it is of great significance to develop a homogenization method that takes the surface effect into account in order to accurately predict the bending deformation behavior of metamaterial beams.

Based on the aforementioned literature, our study primarily addresses the following two issues: first, the ability of metamaterial beams to resist deformation under bending loads is influenced by the surface effect, yet this mechanism remains unclear when scale separation assumptions do not hold. Therefore, it is necessary to investigate the underlying mechanisms of the surface effect during bending deformation of porous polymer beams, which holds significant implications for their performance prediction and structural design. Second, since the infinitesimal assumption inherent in multiscale analysis methods is not applicable to porous polymer beams exhibiting a pronounced surface effect, it leads to substantial computational inaccuracies. Moreover, the lattice constituting porous polymer beams possesses complex microstructural features, such that performing direct numerical simulations results in low computational efficiency and high cost. To address these challenges, we aim to develop an efficient and accurate homogenization method that incorporates the surface effect under bending loads, thereby enabling broader applications of metamaterials in aerospace, civil engineering, and biomedical fields.

**Figure 1:**
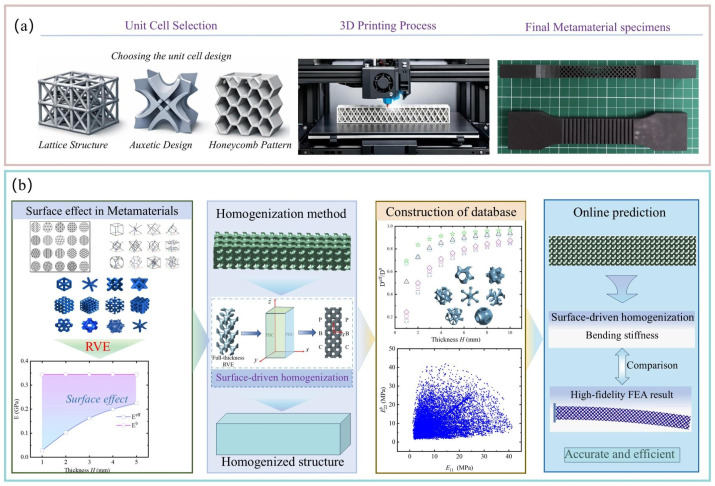
(**a**) Fabrication process of the metamaterial beam using 3D printing. (**b**) A homogenization method incorporating the surface effect for size-dependent bending behavior of porous polymer beams, and *E* represents the elastic modulus and *D* represents the bending stiffness.

The organization of this paper is as follows: Section 2 presents the significant size-dependent bending deformation observed in porous polymer beams and investigates its underlying mechanisms using high-fidelity finite element analysis. In Section 3, an equivalent model for porous polymer beams incorporating surface-driven moment of inertia is developed and an online prediction framework based on the offline dataset generated by the surface-driven homogenization method is established for the size-dependent bending response. Section 4 discusses in detail the relationship between surface effects and microstructural characteristics, and evaluates the accuracy and computational efficiency of the proposed homogenization method. Finally, the main conclusions of the study are summarized in Section 5. Figure 1 presents an overall schematic of the study, which demonstrates the advantages of the homogenization method that accounts for the surface effect, as compared to high-fidelity finite element methods and multi-scale analysis methods that neglect the surface effect. By using this homogenization method to construct a lattice-related database, it becomes possible to accurately and efficiently predict the mechanical deformation behavior of porous polymer beams, which is of significant importance for the structural design and performance prediction of porous polymer beams with complex microstructures.

## 2. Problem Statement

The subject of this study is slender porous polymer beams composed of repeated lattices with complex microstructures, involving four types of lattices: Diamond, Primitive, Fischer-Koch S, and Split P, which can be transformed into solid structures by offsetting triply periodic minimal surfaces (TPMS). Their properties, such as effective elastic modulus and specific stiffness, can be tuned by adjusting porosity, lattice type, and lattice constant. The lattice constant is denoted as *a*, and the porosity as $V_{p}$.

Pronounced surface effect in porous polymer beams composed of TPMS lattices, and Figure 1a illustrates the variation trend of the effective modulus with beam thickness, which was obtained by putting the full resolved microstructures into the high-fidelity finite element simulations. The material used in this study is Photosensitive Resin 9400, with a Young’s modulus of E=2.700GPa, a density of $\rho =1100\;\mathrm{kg}/m^{3}$, and a Poisson’s ratio of ν=0.41. The models used in this study to investigate the influence of surface effects on the overall stiffness of metamaterial beams are all based on the assumption of linear elastic material behavior. The effective elastic modulus increases with the increasing of the macroscopic thickness *H*. When H=5mm, the effective modulus is 7.74 times that of H=1mm, and this trend continues with a further increase in thickness. Meanwhile, the bulk elastic modulus shows a 12-fold increase relative to the configuration in the H=1mm case. This surprising phenomenon exerts a substantial influence on the deformation response of porous polymer beams subjected to bending loads. However, the underlying mechanisms are unclear, which limits its design and prediction.

**Figure 2:**
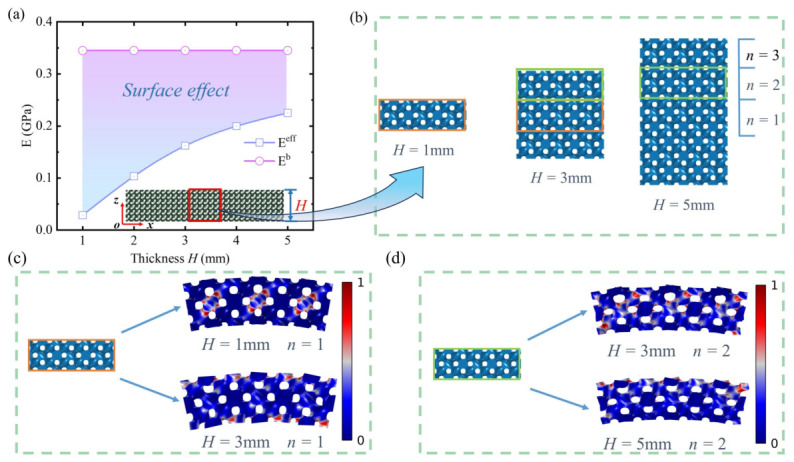
Mechanism of surface effect in porous polymer beams. (**a**) Surface effect of porous polymer beams with different thicknesses. (**b**) Local view of porous polymer beams with varying thicknesses. (**c**) Normalized stress distribution in region n=1 for different beam thicknesses *H*. (**d**) Normalized stress distribution in region n=2 for different beam thicknesses *H*. The color bar in the figure, ranging from 0 to 1, represents the normalized von Mises stress. For each structure, a color closer to 1 indicates a higher stress at that location, and the orange and green boxes indicate regions at the same spatial coordinates under different thickness conditions.

Classical mechanics theory holds that, when resisting bending deformation, the regions farther from the neutral axis contribute more significantly. However, our study finds that, when subjected to loading, the surface regions of porous polymer beams contribute less to resisting bending deformation compared to the core regions, which is determined by the microstructure of the metamaterial beam. Figure 2 illustrates the intrinsic mechanism behind the surface effect in porous polymer beams. Figure 2b shows local views of porous polymer beams with varying thickness *H*, where *n* represents a different layer, and lattices within the same layer undergo identical deformation. As shown in Figure 2c, when H=1mm, in the deformed metamaterial beam, the stress in the layer of n=1mm is mainly concentrated in the central region, indicating that the surface region contributes little to resisting bending deformation. Whereas for the H=3mm beam, the stress in the core layer n=1 after deformation is predominantly concentrated near the surface region. Similarly, as shown in Figure 2d, when n=2, the stress distribution in this region after deformation exhibits a pattern similar to that in Figure 2c for H=3mm compared to H=5mm, stress concentrates in the central region of n=2 at H=3mm, whereas at H=5mm, it concentrates near the surface of n=2, which is determined by the microstructure of the lattice.

**Figure 3:**
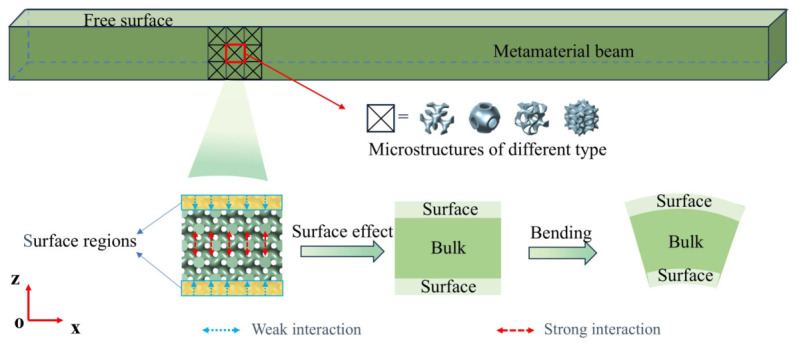
Surface effect of slender metamaterial structures under bending deformation.

Figure 3 presents the modeling of slender porous polymer beams based on the surface effect, where, due to the existence of two free surfaces along the thickness direction of the metamaterial beam, the lattices located at these free surfaces lack interacting structures on the boundary facing the free surface, which is in contrast to the lattices in the middle core region. The lattices in the core region, being surrounded by the microstructure on all sides, can transfer loads to neighboring lattices during bending deformation, whereas the lattices at the surface region experience less constraint than those in the core, and this constraint deficiency results in a weaker ability of the surface region to resist bending deformation. This phenomenon differs from the nanoscale surface effect, which originates from surface atoms having fewer bonding neighbors or lower coordination numbers compared to the bulk atoms [70]. In porous polymer beams with complex microstructures, the surface effect primarily arises because the microstructure in the surface region experiences constraint deficiencies under loading, which is different from the situation in the core region. For TPMS structures, curvature variations within the geometry may also influence the local mechanical response; however, in the present study, this effect is implicitly captured within the geometric representation adopted in the current model and is not treated as an independent variable for analysis. Moreover, in contrast to deformation under pure tensile loading, the surface region of the metamaterial beam, which is located farther from the neutral axis, plays a more significant role when subjected to bending loads, so that the influence of the surface effect becomes more pronounced. Especially when the metamaterial beam is relatively thin, such that the lattice size becomes comparable to the macroscopic thickness, the constraint deficiency caused by the free surface cannot be ignored, since it significantly affects the beam’s ability to resist bending deformation. Therefore, when calculating the bending resistance of porous polymer beams subjected to bending loads, it is necessary to take the surface elasticity of the microstructure into account. However, because the microstructural surfaces suffer from constraint deficiencies, the infinitesimal strain assumption is violated, which causes some classical mechanics-based multiscale analysis methods to become unsuitable. Consequently, it is necessary to develop a homogenization method based on classical mechanics that incorporates the surface effect in order to improve the accuracy of predictions. Under bending loads, the weaker surface regions may be associated with local shear or cell deformation modes. However, the focus of this study is on the overall structural stiffness and the macroscopic surface effects, and due to the limitations of the model, a systematic analysis of local deformation modes has not been conducted. It should also be noted that polymers used in additive manufacturing generally exhibit viscoelastic behavior, meaning their mechanical properties depend on time, loading rate, and temperature, which may further influence the deformation mechanisms at free surfaces. Previous studies have shown that 3D printed polymer samples can exhibit pronounced viscoelastic creep and nonlinear responses under sustained loads, and due to the layer-by-layer fabrication process and directional deposition of material chains, they also display anisotropic mechanical behavior related to the printing orientation [71,72]. These phenomena indicate that viscoelasticity and printing-induced anisotropy may alter local stiffness and strain distributions near free surfaces, thereby modulating surface effects in ways that cannot be captured by purely linear elastic homogenization. However, the present study is based on ideal linear elastic materials, and the effects of polymer viscoelasticity and printing-induced anisotropy on surface effects are not considered. Future work may incorporate viscoelastic constitutive models and anisotropic mechanical responses to more accurately describe the time- and orientation-dependent mechanical behavior of 3D printed polymer metamaterials.

Based on the above description, our research focuses on addressing the following two issues: Firstly, from a scientific perspective, the intrinsic mechanism of the surface effect in porous polymer beams under non-scale separation conditions remains unclear, which limits the accuracy of performance prediction and structural design. Therefore, revealing the intrinsic mechanism of surface effect in porous polymer beams is of great significance. Secondly, from a technical standpoint, DNS for calculating the mechanical properties of porous polymer beams requires substantial computational resources, while the prediction errors of classical homogenization methods based on the scale separation assumption are relatively large, especially when the lattice size composing the metamaterial beam approaches the macroscopic thickness of the beam. Thus, it is necessary to develop an efficient and accurate homogenization method that incorporates the surface effect of the metamaterial beam.

By addressing the aforementioned two issues, the influence characteristics of the surface effect on the bending resistance of porous polymer beams can be obtained, and intrinsic size-dependent parameters of the beams can be derived through finite element analysis, thereby revealing the mechanism by which surface effect caused by the beam’s microstructure affects its mechanical properties.

## 3. Online Prediction Framework Based on the Surface-Driven Homogenization Method

In this section, an online prediction framework based on the offline dataset generated by the surface-driven homogenization method is established for the size-dependent bending response.

### 3.1. Equivalent Model for Porous Polymer Beams Incorporating the Surface-Driven Moment of Inertia

Classical mechanics-based multiscale analysis methods often neglect the surface effect when calculating the bending deformation of porous polymer beams, which frequently leads to significant computational relative errors. When surface effect is taken into account, since the surface regions are located far from the neutral axis, and according to the relationship between bending stiffness and the moment of inertia of the cross-section, the surface regions contribute more significantly to the beam’s resistance to bending deformation. Therefore, the surface effect cannot be ignored in the bending behavior of beams. In this section, in order to accurately characterize the effective stiffness of metamaterial beam structures subjected to bending loads, the moment of inertia is introduced, and a homogenization method incorporating the surface effect of porous polymer beams is developed. Furthermore, a normalized bending stiffness expression related to the structural scale is established, which reflects the influence of the surface effect on the mechanical response of the porous polymer beams.

**Figure 4:**
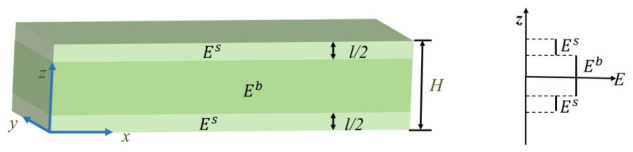
Bulk and surface elastic moduli of the solid beam equivalent to the metamaterial beam.

To describe the influence of surface effect on the bending stiffness of the beam, a size-dependent effective elastic modulus model was established, as shown in Figure 4. Since the surface region of the porous metamaterial beam lacks some interacting structures, the elastic tensors of the surface region and the core region subjected to bending loads are different. In the figure, the porous internal structure of the beam is converted into a solid structure, while surface effects are taken into account. Therefore, along the macroscopic thickness direction, the beam can be divided into two regions: the core region, represented by dark blue, and the surface region, represented by light blue. The surface-affected region can be expressed as $\left(H-l/2\right)\leq \left|z\right|\leq H/2$, where *z* is the thickness direction in the Cartesian coordinate system, and the core region corresponds to $|z|<\left(H-l/2\right)$. It is assumed that the elastic modulus of the surface region is $E^{s}$, while that of the core region (i.e., the bulk modulus) is $E^{b}$. The parameter introduced in this study does not represent a true mechanical property of the surface region in a strict physical sense. Instead, it is an effective parameter used to characterize the influence of surface effects, and its value is determined through the subsequent homogenization procedure. It reflects the contribution of the surface region within the microstructure to the overall mechanical response, and can be regarded as an effective representation of surface effects, thereby effectively capturing their influence on the macroscopic mechanical behavior.

At this point, the effective elastic modulus of different regions of the beam can be expressed as Equation (1):

(1) $$E^{\mathrm{eff}}=\left\{\begin{array}{cc} E^{s}, & (H-l)/2\;\leq \;|z|\;\leq \;H/2\;\\ E^{b}, & |z|<(H-l)/2 \end{array}\right.$$

where $E^{s}$ is the elastic modulus of the surface region, $E^{b}$ is the bulk elastic modulus, *H* is the thickness of the beam, and *l* is the total thickness of the surface region, which depends on the lattice type, porosity, and lattice constant, but is independent of the material type.

According to Figure 3 and Equation (1), the effective elastic modulus of the entire beam is denoted as $E^{\mathrm{eff}}$. The effective bending stiffness of the beam can then be expressed as $E^{\mathrm{eff}}I$. The relationship between $E^{\mathrm{eff}}$, $E^{s}$ and $E^{b}$ can be expressed by Equation (2), where *I* is the total moment of inertia of an equivalent solid beam about the *y*-axis, given by Equation (3):

(2) $$E^{\mathrm{eff}}I=E^{s}I^{s}+E^{b}I^{b}$$

(3) $$I=\frac{WH^{3}}{12}$$

where $I^{b}$ and $I^{s}$ are the moments of inertia about the *y*-axis for the core region and the surface region, respectively. They are given by:

(4) $$I^{b}=\frac{W{\left(H-l\right)}^{3}}{12}$$

(5) $$I^{s}=I-I^{b}$$

The relationship between the elastic modulus of the surface region and the bulk elastic modulus is assumed as Equation (6):

(6) $$E^{s}=kE^{b}$$

where *k* is the surface strength factor. Then, the relationship between the effective elastic modulus and the bulk elastic modulus as a function of thickness *H* can be expressed as follows:

(7) $$\frac{E^{\mathrm{eff}}}{E^{b}}=k+\left(1-k\right){\left(1-\frac{l}{H}\right)}^{3}$$

where *l* is surface thickness. Note that *k* and *l* are influenced by the microstructural features of the lattice and depend solely on the structural properties of the lattice.

The bending stiffness can be used to evaluate the beam’s resistance to bending deformation. After homogenization incorporating surface effect, the effective bending stiffness is $D^{\mathrm{eff}}=E^{\mathrm{eff}}I$, while the bending stiffness homogenized by classical mechanics-based multiscale analysis is $D^{0}=E^{b}I$. The ratio between these two is:

(8) $$\frac{D^{\mathrm{eff}}}{D^{0}}=k+\left(1-k\right){\left(1-\frac{l}{H}\right)}^{3}$$

This dimensionless ratio describes the influence of surface effect on the beam’s effective bending stiffness. To understand the mechanism of surface effect during bending, it is necessary to calibrate the two intrinsic parameters *k* and *l*. However, incorporating fully resolved microstructures into the direct high-fidelity simulation (DHS) requires substantial computational resources and time. Therefore, a surface-driven homogenization method based on representative volume elements (RVEs) is necessary.

### 3.2. Construction of an Online Prediction Framework

The surface-driven online prediction framework requires the construction of a discrete dataset related to lattice dimensions, thereby enabling the online prediction of the mechanical performance of porous polymer beams with different thicknesses.

**Figure 5:**
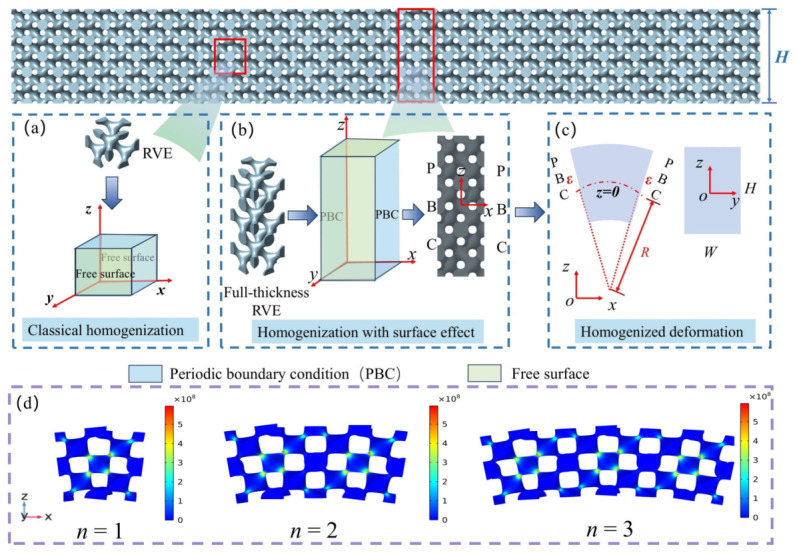
Boundary value problems of two different homogenization methods. (**a**) Classical homogenization method based on the lattice. (**b**) Homogenization method incorporating surface effect based on the full-thickness RVE. (**c**) Deformation of the homogenized structure. (**d**) Effect of RVE size (i.e., the number of unit cells it contains) on the results: analysis of the influence of RVEs composed of 1, 2, and 3 unit cells, respectively, with periodic boundary conditions applied in the x-direction, the color bar in the figure represents the von Mises stress, with units of Pa.

A critical aspect of the homogenization method is the selection of a representative volume element (RVE), which exhibits periodic structural characteristics at the microscale, from within a macroscopically non-uniform structure. Through appropriate computation and analysis of the RVE, its mechanical behavior at the microscale can be obtained, thereby allowing the macroscopic performance of the entire structure to be accurately predicted. In this study, the macroscale non-uniform structure is a complex beam, from which a representative volume element (RVE) needs to be extracted to calculate the constitutive parameters of the non-uniform material. The homogenization method based on classical mechanics and multiscale analysis does not incorporate the surface effect and is used to obtain the bulk elastic modulus $E^{b}$. This method relies on the infinitesimal strain assumption, which requires that the microscale of the structure be much smaller than the macroscale [30]. Therefore, in order to meet this requirement, periodic boundary conditions must be applied at the lattice boundaries of interest. Since this work focuses on the bending behavior of beams within the xz-plane, it is only necessary to ensure that the boundaries of the lattice in the *x* and *z* directions satisfy periodic boundary conditions, as shown in Figure 5a. During bending, the lattice is essentially subjected to a combination of tension and compression in the *x* direction, while the periodic boundary condition in the *z* direction is only introduced to eliminate the influence of the surface effect along the thickness direction.

Thus, when calculating the bulk modulus in the *x*-direction, imposing a kinematically uniform boundary condition with a prescribed displacement perturbation along *x*, while allowing the transverse (*z*) direction to be traction-free. Equivalently, periodic boundary conditions can be used to satisfy the Hill–Mandel condition [69,73].

Let $V=V_{m}\cup V_{p}$ be the RVE volume, where $V_{m}$ and $V_{p}$ denote the matrix (solid) and pore (void) subdomains, respectively, and $V=V_{m}+V_{p}$. The macroscopic (volume-averaged) strain and stress are defined as follows:

(9) $$\bar{\varepsilon }=\frac{1}{V}\int_{V}\varepsilon \;\chi_{m}\;dV=\frac{V_{m}}{V}\left(\frac{1}{V_{m}}\int_{V_{m}}\varepsilon \;dV\right),$$

(10) $$\bar{\sigma }=\frac{1}{V}\int_{V}\sigma \;\chi_{m}\;dV=\frac{1}{V}\int_{V_{m}}\sigma \;dV,$$

where $\chi_{m}$ is the indicator function of the matrix phase ($\chi_{m}=1$ in $V_{m}$, 0 in $V_{p}$), stresses in the void phase vanish. The effective constitutive relation reads:

(11) $$\bar{\sigma }=C^{b}:\bar{\varepsilon },$$

where $C^{b}$ is the effective elastic tensor obtained from the multiscale analysis. For uniaxial homogenization along *x*, we extract $E_{x}^{b}={\bar{\sigma }}_{xx}/{\bar{\varepsilon }}_{xx}\;\mathrm{under}\;{\bar{\varepsilon }}_{xx}\neq 0$.

However, at the microscale, the material’s microstructure is inherently non-uniform, and when it is subjected to external loads, the internal load transfer paths become highly complex. Moreover, due to the unique curved geometry of triply periodic minimal surfaces (TPMS), the connections between surface regions tend to exhibit certain structural imperfections compared to those within the core region, which consequently leads to a reduction in the effective elastic modulus. In addition, as the total thickness of the beam becomes thinner, the surface effect induced by these surface regions becomes increasingly prominent. In such circumstances, the structural configuration no longer satisfies the infinitesimal assumption required by classical multiscale theory. Therefore, theoretical multiscale analysis is no longer suitable for predicting the mechanical behavior of microstructures with pronounced surface effects. To improve the accuracy of predicting the material’s macroscopic mechanical properties, a homogenization method that accounts for surface effects is proposed, so as to ensure equivalence with the actual microstructure.

As shown in Figure 5b, in order to investigate the influence of thickness on the surface effect, it is first necessary to ensure that the thickness in the *z*-direction remains uncertain. Subsequently, a representative volume element (RVE) with full thickness is selected, where periodic boundary conditions are applied to both boundaries along the *x*-direction, so as to simulate a beam model whose length scale is significantly larger than its thickness. This configuration also ensures that the boundaries satisfy both displacement continuity and traction continuity conditions. Similarly, since the analysis focuses solely on the bending deformation of the beam within the x–z plane, the boundaries in the *y*-direction are treated as free surfaces. The thickness of the beam varies along the *z* direction; therefore, the free surface is defined in this manner, while periodic boundary conditions are applied to the RVE along the beam length direction. This allows the RVE to effectively capture the influence of thickness variations on local stiffness and surface effects. Although the RVE represents a local unit, the global boundary conditions of the actual beam structure do not alter the physical meaning or the local mechanical response of the free surface along the *z* direction. Based on these conditions, the effective elastic modulus $E^{\mathrm{eff}}$ of the full-thickness RVE subjected to bending deformation can be obtained, which is used to construct an offline database correlated to different lattice configurations. Because periodic boundary conditions are imposed along the *x*-direction in order to simulate an infinitely long metamaterial beam, and given that the thickness scale is much smaller than the length scale, the Euler–Bernoulli beam theory is adopted for the analysis. The lattice is subjected to the strain field described in Equation (12) at the *x*-direction boundaries, thereby inducing bending deformation, as illustrated in Figure 5c. In addition, to verify the rationality of the RVE selected in this study, RVEs composed of 1, 2, and 3 unit cells were constructed, respectively, and periodic boundary conditions were applied in the *x*-direction for comparison, as shown in Figure 5d, with a porosity of 0.9. The results show that under the same strain conditions, the deformation patterns and stress distributions of the unit cells remain essentially consistent for different RVE sizes. Further calculations indicate that the effective elastic moduli obtained from the three representative volume elements (RVEs) are identical, demonstrating that the number of unit cells contained in the RVE has no effect on the results of this study. This confirms that the RVE size selected in this study has sufficient representativeness and convergence, and can reliably predict the macroscopic stiffness of the structure as well as the trend of surface effects.

(12) $$\varepsilon =\frac{z}{R}$$

where *R* is the radius of curvature.

Then, according to the formula for the lattice moment of inertia about the *y*-axis, Equation (13), the moment of inertia *I* can be determined as follows:

(13) $$I=\int_{A}z^{2}\;dA$$

However, when incorporating the lattice microstructure, the moment of inertia *I* is not easy to obtain. Based on Figure 5b, the TPMS with porous microstructure is equivalent to a homogeneous model, and its cross-section is approximated as a rectangular section, as shown in Figure 5c. At this point, the homogenized moment of inertia $I^{\prime }$ about the y-axis can be easily obtained, expressed by Equation (14):

(14) $$I^{\prime }=\frac{WH^{3}}{12}$$

where $I^{\prime }$ is the homogenized moment of inertia about the *y*-axis, and *W* and *H* are the width and height of the cross-section, respectively.

At this stage, the relationship between the bending moment *M* at the *x*-boundary and the homogenized effective elastic modulus can be obtained according to Equation (15):

(15) $$M=\frac{E^{\mathrm{eff}}I^{\prime }}{R}$$

By using a surface-driven homogenization method, the effective elastic modulus of porous polymer beams with different thicknesses can be obtained. It is worth noting that, unlike classical multiscale analysis, this surface-effect-informed homogenization method considers the discontinuity of the medium in the thickness direction at the TPMS microstructure surface. Therefore, when calculating $E^{\mathrm{eff}}$, it is necessary to ensure that the surfaces along the *z*-direction are free, which is exactly the opposite of the condition for calculating $E^{b}$. The calculation of $E^{b}$ does not incorporate the surface effect and still satisfies the infinitesimal assumption, so periodic boundary conditions must be imposed along the *z*-direction as well.

**Figure 6:**
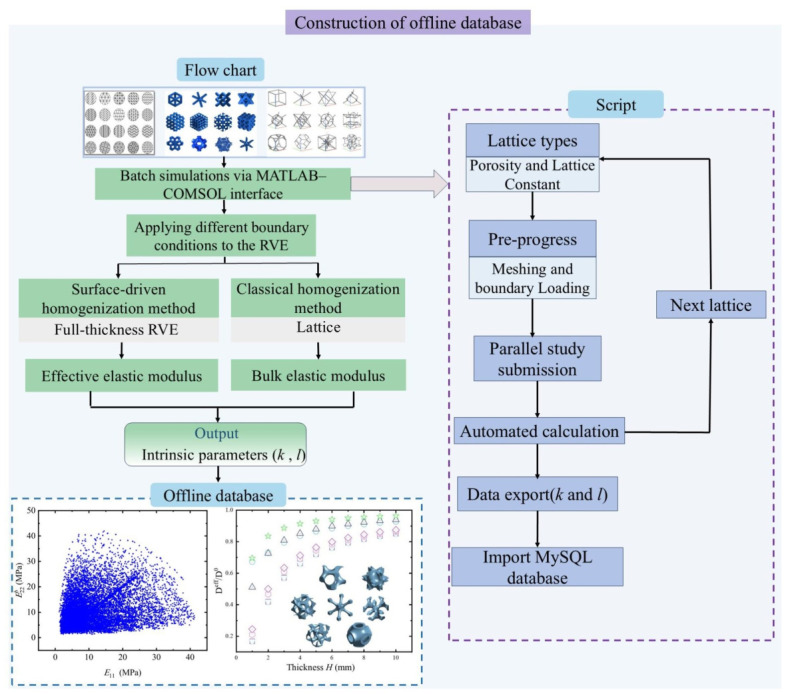
Construction of the offline database by the surface-driven homogenization method.

For different types of lattices, the surface thickness *l* and surface strength factor *k* can be obtained using the above homogenization method. In this study, the parameters *k* and *l* jointly influence the overall stiffness of the macroscopic beam and the surface effect; however, they are physically independent. Both parameters are determined through numerical calibration to ensure that the model can accurately predict the macroscopic structural response. Within the scope of the present study, no direct mathematical relationship between *k* and *l* is assumed. Then, a lattice-related database composed of *k* and *l* can be established, as shown in Figure 6. By constructing this offline database, it is possible to realize online prediction of the mechanical deformation behavior of porous polymer beams filled with different lattices subjected to bending loads in an accurate and efficient manner. It should be noted that, in this study, *k* and *l* are calibrated based on the error-minimization criterion. Specifically, once the geometric parameters of the lattice are determined, the proposed homogenization model exhibits a uniquely optimal set of *k* and *l* values that best represent the true mechanical response of the beam.

## 4. Results and Discussion

In this section, the validity of the expression for the effective bending stiffness based on the surface effect, Equation (8) is confirmed. The influences of the TPMS lattice type, porosity, and lattice constant on the surface strength factor *k* and surface thickness *l*, as well as their coupled effects on the bending stiffness of the metamaterial beam, are discussed. Finally, by constructing porous polymer beams with complex microstructures, the computational accuracy and efficiency of the proposed homogenization method are evaluated. For convenience, the normalized bending stiffness $D^{\mathrm{eff}}/D^{0}$ is used to characterize the variation trend of the beam’s effective bending stiffness with the surface effect at different thicknesses. In the present study, the employed model assumes the material to be homogeneous and defect-free, and does not take into account potential manufacturing errors during 3D printing, such as material defects, porosity, or residual stresses.

### 4.1. Surface-Driven Size-Dependent Bending Stiffness

**Figure 7:**
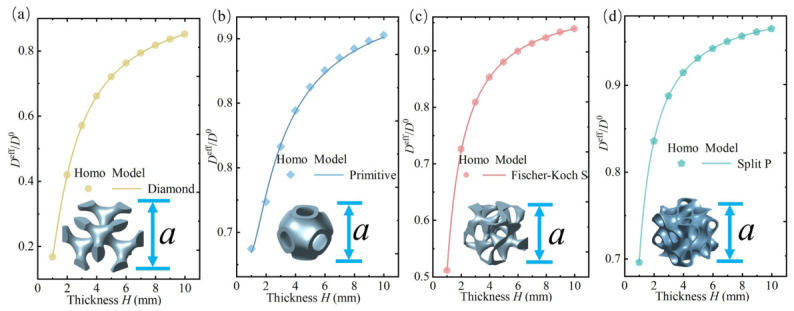
Normalized bending stiffness of porous polymer beams composed of different lattice types. (**a**) The Diamond, (**b**) Primitive, (**c**) Fischer-Koch S, and (**d**) Split P models refer to the models as expressed in (8), *a* represents the lattice constant. Homo refers to the surface-driven homogenization method.

Taking the Diamond lattice as an example, the coupled effects of the intrinsic parameters *k* and *l* on the effective bending stiffness of the beam at different thicknesses are investigated. In the calculations, the lattice constant is set as a=1mm, and the porosity $V_{p}=0.8$. By increasing the number of lattice units along the *z*-direction, the thickness of the metamaterial beam is varied, and the variation trend of the normalized bending stiffness $D^{\mathrm{eff}}/D^{0}$ and thickness is obtained, thus quantifying the impact of the surface effect on the effective bending stiffness of the beam at different thicknesses. The results indicate that the beam’s effective bending stiffness is significantly influenced by surface effect, which has the greatest impact when the thickness is 1 mm. As shown in Figure 7a, $D^{\mathrm{eff}}/D^{0}$ is only 16.7%, which means that the bending stiffness obtained by the homogenization method without incorporating the surface effect is six times that obtained when the surface effect is included. Therefore, studying the influence of the surface effect on the bending stiffness of the beam is extremely necessary. However, it can also be observed that as the thickness increases continuously, the ratio $D^{\mathrm{eff}}/D^{0}$ increases correspondingly, indicating that the influence of the surface effect on the beam’s bending stiffness diminishes with increasing thickness. Next, the mechanism behind the observed trend of normalized bending stiffness changing with thickness will be explained, using the Diamond lattice as a specific example.

The normalized bending stiffness $D^{\mathrm{eff}}/D^{0}$ increases continuously with the increase in thickness, which indicates that the region influenced by the surface effect exhibits an overall stiffness softening effect compared to the core region. This can also be understood from the two intrinsic parameters *k* and *l* in Equation (8). The calculation results show that the surface strength factor *k* is less than 1, indicating that the elastic modulus of the surface region is lower than that of the core region. Therefore, the effective bending stiffness of the beam incorporating the surface effect exhibits a softening behavior. Furthermore, as the thickness increases, the influence of the surface effect on the bending stiffness gradually decreases. This is because when the thickness is small, the ratio of the surface thickness *l* to the macroscopic thickness is relatively large, leading to a more significant impact on the effective bending stiffness. However, as the thickness continues to increase, this ratio decreases, resulting in a diminished contribution of the surface effect to the effective bending stiffness. Consequently, the observed stiffness softening phenomenon becomes progressively weaker.

The above analysis indicates that the intensity of the surface effect depends on the microstructure of the surface region, which determines the values of the two intrinsic parameters, *k* and *l*, when the beam undergoes bending. The coupled interaction between *k* and *l* governs the magnitude of the surface effect on the effective bending stiffness. Notably, when the macroscopic thickness of the beam approaches the lattice size, the surface effect becomes particularly significant. It is worth noting that the present study was conducted based on the linear elastic response of the material. When material nonlinear behavior is considered, under higher strains or larger loads, nonlinear effects will inevitably influence the mechanical behavior of the surface region. In this study, nonlinear factors are not included within the scope of the model.

### 4.2. Microstructure-Dependent Surface Effect

Both theoretical analysis and numerical simulations indicate that the magnitude of the surface effect is related to the microscale structure of the lattice, which in TPMS depends on the lattice type, porosity, and lattice constant. This section investigates the variation of surface effect by altering the lattice microscale features, while clarifying the contributions of the intrinsic parameters *k* and *l* to the surface effect with different types of lattices, porosities, and lattice constants. Furthermore, it elucidates the underlying mechanism by which these parameters influence the effective bending stiffness of the beam.

First, the surface effect subjected to bending of four different lattice types was studied, with the lattice constant a=1mm and porosity $V_{p}=0.8$. The variation trends of normalized bending stiffness with thickness for different lattice types were obtained, as shown in Figure 7a–d. Among the four TPMS lattices studied, the Diamond lattice exhibits the most significant surface effect: when the thickness is 1 mm, the normalized bending stiffness $D^{\mathrm{eff}}/D^{0}$ is 16.7%, and it increases to 85% at 10 mm thickness. Next are the Fischer–Koch S and Primitive lattices, where $D^{\mathrm{eff}}/D^{0}$ are 51% and 65% respectively at 1 mm thickness, while the Split P lattice reaches 70%. It is noteworthy that, except for the Diamond lattice, the other three lattices have normalized bending stiffness values above 90% at 10 mm thickness, indicating that the surface effect’s influence on effective bending stiffness becomes very small at this scale. Compared to the other three lattices, the Diamond lattice exhibits a stronger surface effect. To explain this, a qualitative interpretation is provided from a microscale perspective: compared to its core region, the surface microstructure is more dispersed, resulting in a lack of effective load-carrying paths under bending. Consequently, the surface region is more prone to local deformation, leading to a more pronounced surface stiffness softening effect. This description is intended to provide a physical explanation for the reduction in macroscopic beam stiffness.

In contrast, the other three lattices have relatively tighter connections in their surface microstructures, which limit deformation subjected to bending loads. Consequently, although surface stiffness softening still exists, it is much less significant compared to the Diamond lattice. Particularly, the Split P lattice has almost no isolated surface regions, explaining why the stiffness softening phenomenon is weakest among the four lattice types.

**Figure 8:**
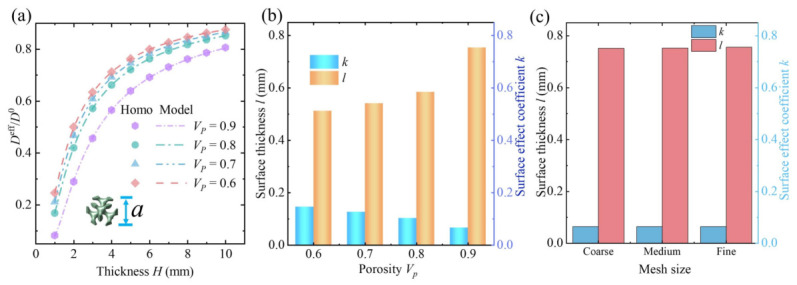
Effects of porosity on the normalized bending stiffness of porous polymer beams. (**a**) Normalized bending stiffness of beams with different porosities, *a* represents the lattice constant. (**b**) Surface thickness *l* and surface strength factor *k* with different porosities. Model refers to the model as expressed in (8). Homo refers to the surface-driven homogenization method. (**c**) Sensitivity analysis of the intrinsic parameters *k* and *l* with respect to mesh density, using a Diamond lattice with a porosity of 0.9 and a lattice constant of 1 mm, with *h* highlighted.

Secondly, The Diamond lattice is used as an example to investigate the influence mechanisms of intrinsic parameters on effective bending stiffness under varying porosity conditions. Four Diamond lattices with porosities of 0.6, 0.7, 0.8, and 0.9 are set respectively, in order to observe the variation patterns of intrinsic parameters and surface effect. The influence on the normalized bending stiffness $D^{\mathrm{eff}}/D^{0}$ is shown in Figure 8a. It can be seen that, as the porosity decreases, the influence of surface effect on the normalized bending stiffness also becomes increasingly smaller. When the thickness is 1 mm, as the porosity decreases from 0.9 to 0.6, the normalized bending stiffness increases from 8.16% to 24.5%, which indicates that, for the same type of lattice, the larger the porosity is, the more significant the surface effect becomes on the normalized stiffness. When analyzing the effect of porosity on the surface effect, we do not directly use the absolute change in global stiffness to assess its magnitude. Instead, the relative measure $D^{\mathrm{eff}}/D^{0}$ is adopted to characterize the surface effect. As shown in Figure 8a, for the same beam thickness, a higher porosity corresponds to a more pronounced surface effect. In Figure 8b, it can also be observed that the intrinsic parameter *k* decreases with increasing porosity, while *l* exhibits the opposite trend, indicating that the relative stiffness softening of the surface becomes more significant. The increase in surface effect with porosity is a relative change and does not contradict the decrease in overall stiffness. By introducing this relative metric, we can effectively distinguish the contribution of the surface effect from the reduction in global stiffness. It is worth noting that, when the porosity is 0.9, for the Diamond lattice, and when the thickness is 1mm, the bending stiffness obtained by the classical homogenization method is 12 times greater than that obtained by the surface-effect-aware homogenization method. This finding indicates that, under bending deformation, the deflection ratio between beam responses obtained from the two homogenization methods can reach a magnitude of 12, highlighting a substantial discrepancy between the approaches, which is undoubtedly in contradiction with classical mechanical intuition.

Figure 8b also illustrates the variation trends of the surface thickness *l* and the surface strength factor *k* of the Diamond lattice with different porosities. It can be observed that, as the porosity decreases, the surface strength factor *k* gradually increases, while the surface thickness *l* continuously decreases. This indicates that, with the reduction in porosity, the region affected by the surface effect becomes smaller and smaller, and the stiffness of the surface region increases progressively, resulting in a diminishing overall stiffness-softening effect of the lattice. These findings suggest that, for the same type of lattice, reducing the porosity can effectively weaken the influence of the surface effect on the effective bending stiffness. Moreover, we have conducted a systematic sensitivity analysis of the intrinsic parameters *k* and *l* with respect to the mesh density used in the finite element simulations. In the numerical simulations, three different mesh sizes (coarse, medium, and fine) were considered to evaluate their influence on the calibrated parameters. As shown in Figure 8c, the identified values of *k* and *l* remain highly consistent under different mesh discretizations, demonstrating that the proposed calibration method exhibits good robustness and mesh independence.

**Figure 9:**
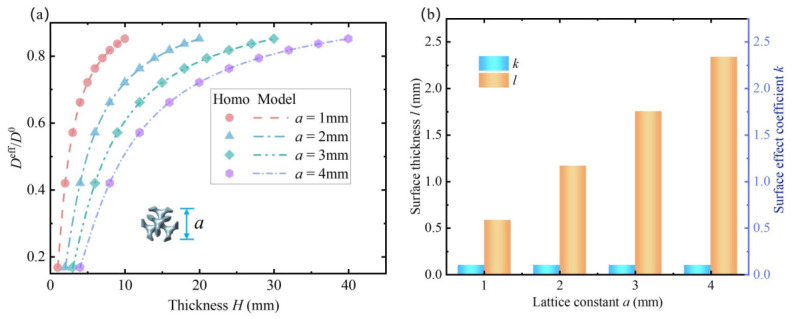
Effect of lattice constant on the normalized bending stiffness of porous polymer beams: (**a**) Normalized bending stiffness of metamaterial beam with different lattice constants. (**b**) Surface layer thickness *l* and surface stiffness factor *k* corresponding to different lattice constants. Model refers to the model as expressed in (8). Homo refers to the surface-driven homogenization method.

In addition, the influence of the lattice constant on the surface effect was also investigated, with the Diamond lattice still being the subject of study. By setting the lattice constant to 1 mm, 2 mm, 3 mm, and 4 mm respectively, the resulting normalized bending stiffness is shown in Figure 9. As the lattice constant increases, the surface thickness *l* continues to increase, indicating that the size-dependent behavior of the lattice becomes more pronounced. However, although the lattice constant keeps increasing, the surface strength factor *k* remains unchanged, because *k* is used to characterize the ratio between the elastic modulus of the surface region and that of the bulk material. Variations in the lattice constant only change the macroscopic geometric scale, while the local microstructural features of the lattice remain unchanged. Therefore, *k* does not vary with the lattice constant. This indicates that, within the scope of the present study, *k* can be regarded as an effective descriptor of the lattice microstructural characteristics and exhibits scale independence.

The above analysis indicates that the surface effect of porous polymer beams subjected to bending loads is influenced not only by the lattice type but also by the porosity and lattice constant. Moreover, increasing the beam thickness, decreasing the porosity, and reducing the lattice constant can all weaken the surface effect. The fundamental reason behind this is that these microscopic characteristics of the metamaterial beam affect the two intrinsic parameters, *k* and *l*, which together determine the contribution of the surface effect to the effective bending stiffness.

Table 1 presents the bulk modulus $E^{b}$ corresponding to different lattice types, lattice constants and porosities, along with the calibrated intrinsic parameters *k* and *l*. As discussed earlier, *k* and *l* exhibit a coupling effect; when *k* is smaller and *l* is larger, it indicates a more pronounced stiffness softening effect in the surface region of the lattice, thereby exerting a greater influence on the effective bending stiffness. This conclusion is reflected in Equation (8), and the intrinsic parameters *k* and *l* of the Diamond lattice in Table 1 further validate this inference. Moreover, the results show that the effects of *k* and *l* on the surface behavior are not independent, but rather coupled. Therefore, it is insufficient to assess the influence of surface effect on the effective bending stiffness based on only one of these intrinsic parameters. Both parameters must be considered simultaneously to accurately evaluate the degree of surface stiffness softening and its impact on the overall mechanical behavior of the metamaterial beam. In this study, the proposed model assumes that the surface region is homogeneous and defect-free, and the surface strength factor *k* is determined through numerical calibration under idealized conditions. Under this assumption, *k* is a dimensionless parameter whose value is independent of the material properties and reflects the contribution of microstructural confinement to surface stiffness. However, during actual 3D printing, defects such as surface roughness, incomplete curing, pores, or residual stresses may occur. These factors can locally alter the mechanical performance of the surface region at the microscale, thereby affecting surface stiffness. Nevertheless, if such defects are relatively uniformly distributed throughout the structure, their impact on *k* is limited and does not significantly change the model’s prediction of the macroscopic beam stiffness.

### 4.3. Accuracy and Efficiency of the Surface-Driven Homogenization Method

**Figure 10:**
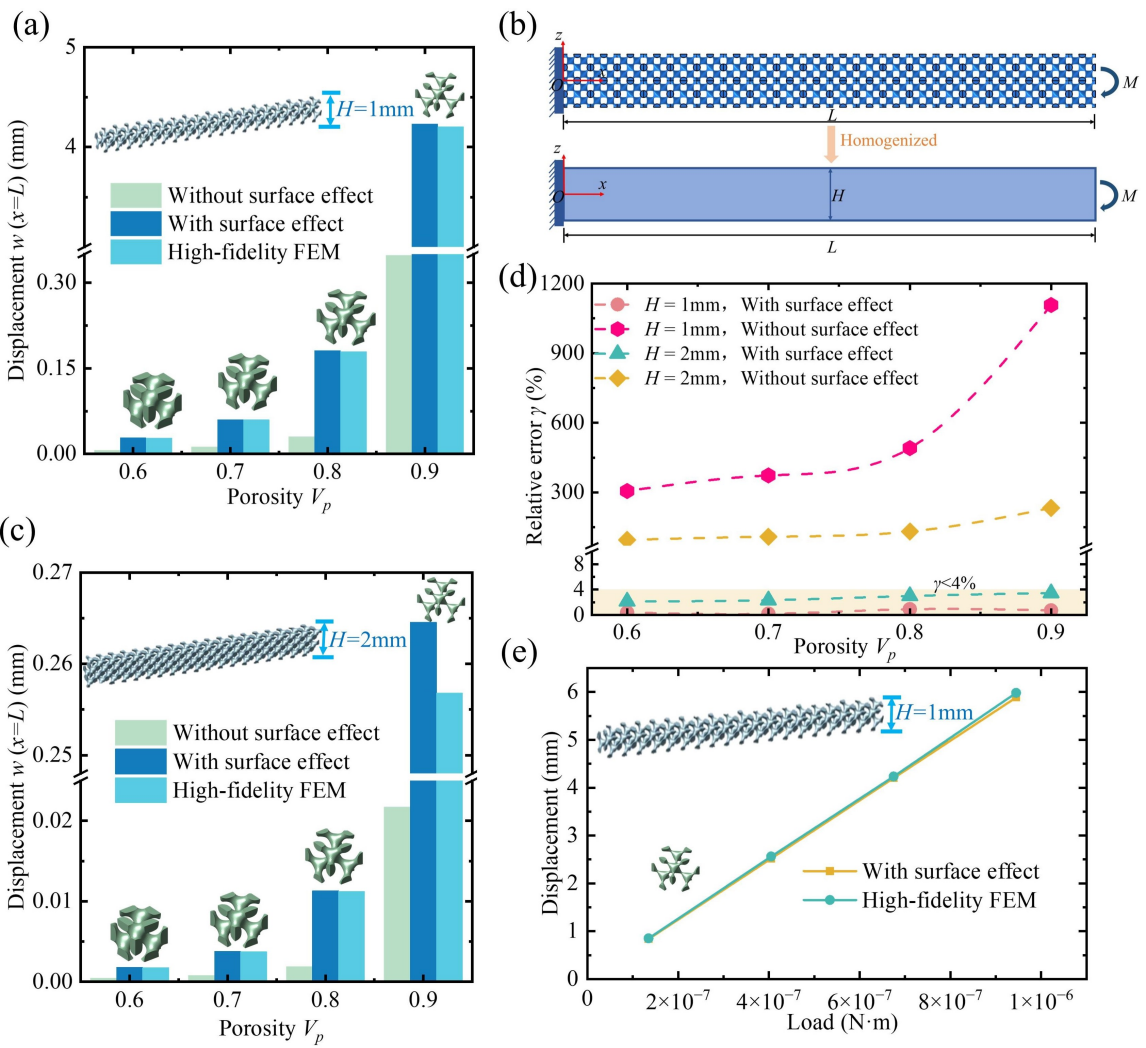
Comparison of end displacements for porous polymer beams with different thicknesses. (**a**) End displacements of the metamaterial cantilever beam with thickness H=1mm. (**b**) Metamaterials subjected to bending loads and their homogenized cantilever Euler-Bernoulli beam model. (**c**) End displacements of the metamaterial cantilever beam with thickness H=2mm. (**d**) Comparison of computational errors of different homogenization methods relative to high-fidelity finite element analysis. (**e**) Load–deflection curves of the beam under different loading conditions, with a porosity of 0.9, a beam length of 20 mm, and a height of 1 mm.

In the previous two parts, the mechanism of the surface effect acting on porous polymer beams under bending deformation, as well as the coupled influence of the intrinsic parameters *k* and *l*, were thoroughly analyzed. Based on this, a homogenization method that takes the surface effect into account was proposed, which can predict the deformation behavior of porous polymer beams subjected to bending loads. In this section, as shown in Figure 10b, a homogenized Euler–Bernoulli beam model, incorporating the surface effect, is established to predict the mechanical response of the beam during bending. The beam’s width and thickness are denoted by *W* and *H*, respectively, and according to the Euler–Bernoulli beam theory, the length-to-thickness ratio L/H should satisfy the condition of being greater than or equal to 10. By applying the homogenization method that incorporates the surface effect, the moment of inertia *I* of the metamaterial beam can be calculated using Equation (14).

According to the Euler–Bernoulli beam theory, the displacement fields of the metamaterial beam in the *x* and *z* directions can be expressed as follows:

(16) $$u_{x}=-z\frac{\partial u_{z}\left(x\right)}{\partial x},\;u_{z}=u_{z}\left(x\right)$$

where $u_{x}$ and $u_{z}$ represent the displacement fields in the *x* and *z* directions, respectively, and $u_{z}\left(x\right)$ denotes the displacement of the neutral axis in the *z* direction. The strain in the *x* direction can be written as follows:

(17) $$\varepsilon_{x}=\frac{\partial u_{x}}{\partial x}=-z\frac{d^{2}u_{z}\left(x\right)}{dx^{2}}$$

Thus, the curvature κ of the metamaterial beam after bending deformation can be represented by:

(18) $$\kappa =\frac{d^{2}u_{z}\left(x\right)}{dx^{2}}$$

By utilizing the relation between the effective bending stiffness and the classical bending stiffness given in Equation (8), the expression for the effective bending stiffness can be derived as follows:

(19) $$D^{\mathrm{eff}}=I\left[kE^{b}+E^{b}\left(1-k\right){\left(1-l/H\right)}^{3}\right]$$

where $E^{b}$ is a known parameter. According to the Euler–Bernoulli beam theory, the equilibrium equation can be formulated as follows:

(20) $$\frac{M}{D_{\mathrm{eff}}}=\frac{d^{2}u_{z}\left(x\right)}{dx^{2}}$$

where *M* denotes the bending moment applied to the beam.

By applying a fixed bending moment *M* at the free end of a cantilever beam, and using the homogenization method incorporating surface effect proposed in this work to predict its bending response, the boundary conditions of the Euler–Bernoulli cantilever beam can be expressed as follows:

(21) $$u_{z}\left(0\right)=0,\;\frac{\partial u_{z}}{\partial x}\left(0\right)=0$$

(22) $$D^{\mathrm{eff}}\frac{d^{2}u_{z}\left(L\right)}{dx^{2}}=M$$

From these, the deflection solution of the beam can be obtained as follows:

(23) $$u_{z}\left(x\right)=-\frac{Mx^{2}}{2D^{\mathrm{eff}}}$$

where the maximum deflection of the beam at x=L is given by:

(24) $$u_{z}^{\max}=-\frac{ML^{2}}{2D^{\mathrm{eff}}}$$

Simultaneously, taking the Diamond lattice as a representative example, a metamaterial beam with a complex microstructure was constructed, as shown in Figure 10b, which still satisfies the Euler–Bernoulli beam theory. If the beam thickness increases or shear effects become significant, the assumptions of the Euler–Bernoulli beam theory may no longer hold, and the applicability and accuracy of the model could be affected. Similarly, a bending moment was applied at the beam’s free end, and a high-fidelity finite element method was employed to study the displacement at the beam’s end subjected to bending load. The results from the high-fidelity finite element analysis were then compared with the predictions from the proposed homogenization method incorporating the surface effect, thereby demonstrating the computational accuracy of the method.

To enhance the reliability of the study, two groups of metamaterial cantilever beams with different macroscopic thicknesses were set up, namely H=a and H=2a, where the lattice constant a=1mm and the beam length L=20mm. For each group, four different porosities of 0.6, 0.7, 0.8, and 0.9 were assigned to demonstrate the effects of macroscopic thickness and lattice porosity on the surface effect of the metamaterial cantilever beams. A bending moment of $M=6.75\times 10^{-7}\;N\cdot m$ was applied at the free end of the cantilever beam. The end displacement of the beam was obtained using the classical multiscale homogenization method, the homogenization method with the surface effect, and the high-fidelity finite element method.

As shown in Figure 10, the results indicate that the surface effect in porous polymer beams under bending deformation is very significant. Compared with the high-fidelity finite element results, the classical mechanics-based multiscale homogenization method is no longer applicable. From Figure 10a,c, it can be seen that subjected to the bending moment *M*, whether the thickness *H* is 1 mm or 2 mm, the end displacement predicted by the classical homogenization method deviates significantly from the high-fidelity finite element calculations, whereas the results predicted by the homogenization method incorporating the surface effect proposed in this work are in excellent agreement with the high-fidelity finite element results. Especially when the porosity is large, as shown in Figure 10a, when $V_{p}=0.9$ and the thickness H=1mm, the classical homogenization method predicts a displacement of 0.348 mm, while the homogenization method proposed here predicts a displacement of 4.202 mm, which matches well with the high-fidelity finite element result of 4.233 mm, and is 12 times larger than the classical homogenization prediction. This clearly demonstrates that the influence of surface effect on the metamaterial beam’s resistance to bending deformation cannot be ignored.

To more intuitively illustrate the impact of surface effect, the bending stiffnesses calculated by the classical multiscale homogenization method, the homogenization method with surface effect, and the high-fidelity finite element method are denoted as $D^{0}$, $D^{\mathrm{eff}}$, and $D^{\mathrm{FEM}}$, respectively. Taking $D^{\mathrm{FEM}}$ as the reference true value, their relative errors are calculated by the following expressions:

(25) $$\gamma_{D^{0}}=\frac{|D^{0}-D^{\mathrm{FEM}}|}{D^{\mathrm{FEM}}}\times 100\%$$

(26) $$\gamma_{D^{\mathrm{eff}}}=\frac{|D^{\mathrm{eff}}-D^{\mathrm{FEM}}|}{D^{\mathrm{FEM}}}\times 100\%$$

The results are shown in Figure 10d. From the figure, it can be observed that the relative error γ of the bending stiffness calculated by the homogenization method that does not incorporate the surface effect is extremely large. When $V_{p}=0.9$, the minimum error reaches 233%, and the maximum error goes up to 1108%, which far exceeds the allowable error range. In contrast, the relative error γ of the bending stiffness obtained by the homogenization method with the surface effect is always below 4%, falling within the permissible error range. This indicates that the predictions made by this homogenization method are reliable. It should be noted that the model proposed in this study is based on the Euler–Bernoulli beam theory, which assumes that the beam cross-section remains planar and perpendicular to the neutral axis during bending, thereby neglecting transverse shear deformation. When the beam thickness increases further, shear effects may become significant, potentially leading to large discrepancies between the model predictions and the actual mechanical response. For thick beams or cases where shear effects are pronounced, Timoshenko beam theory or other modeling approaches that account for shear deformation may be required to maintain prediction accuracy. However, under the requirement of relative error below 4%, as shown in Figure 10d, the present model is highly applicable for beams with H/a≤2, where bending is dominant and the Euler–Bernoulli beam assumptions remain valid.

**Figure 11:**
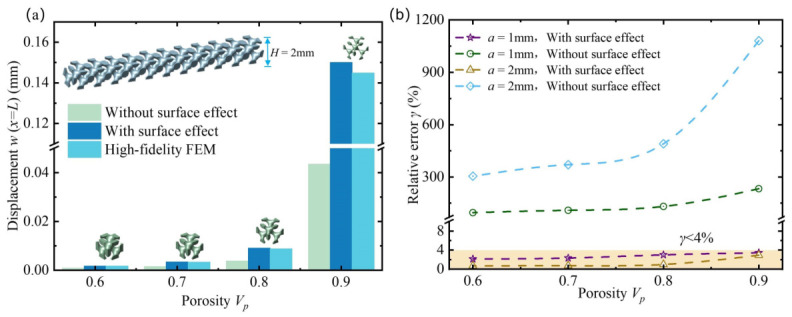
Comparison of end displacements for porous polymer beams with different lattice constants. (**a**) End displacements of the metamaterial beam whose lattice constant a=2mm. (**b**) Comparison of relative errors calculated by different homogenization methods.

Additionally, the influence of the lattice constant on the surface effect of metamaterial cantilever beams was discussed to further verify the accuracy of the homogenization method with surface effect. Two sets of beams were constructed with lattice constants *a* = 1 mm and *a* = 2 mm. The macroscopic thickness H=2mm and length L=20mm of the beams remained unchanged, and four porosity levels were set for each group. The results for a=1mm were taken from Figure 10a. A fixed bending moment $M=6.75\times 10^{-7}\;N\cdot m$ was applied at the beam end, and the relative errors of the end displacement and bending stiffness were obtained, as shown in Figure 11b. The results indicate that, for different lattice constants, the predictions made by the homogenization method with surface effect align well with the high-fidelity finite element results, with errors all below 4%, whereas the classical homogenization method exhibits significantly larger errors. Figure 10e presents the load–deflection curve used to evaluate the equivalent bending stiffness. Since this study is conducted under the assumption of linear elastic material behavior, the calculated deflection exhibits a linear relationship with the applied load. Moreover, under different loading levels, the results obtained from the high-fidelity finite element method are in excellent agreement with those predicted by the proposed homogenization approach, indicating that the proposed model can reliably predict the mechanical response and surface effects of the macroscopic beam.

To demonstrate that the homogenization method proposed in this paper can significantly improve computational efficiency while maintaining high accuracy, a comparison was made between the accuracy and computational time of the high-fidelity finite element method and the homogenization method. The metamaterial beam is composed of Diamond lattice cells, with *L* and *H* representing the macroscopic length and thickness of the beam, respectively. The lattice constant is set as a=1mm, and the porosity is $V_{p}=0.9$. The boundary conditions and loading are consistent with those described previously. The displacement at the beam’s end was calculated and compared, with the results presented in Table 2.

All simulations were conducted on a high-performance workstation equipped with two AMD EPYC 7502 processors (each with 32 cores and a clock speed of 2.50 GHz) and 128 GB of memory, utilizing shared memory parallel computing during the simulation process. As shown in Table 2, the surface-driven homogenization method not only ensures high computational accuracy but also significantly reduces computation time. The efficiency gain is attributed to the rich microstructural details present in the metamaterial beam, and model recognition and processing consume the majority of the numerical simulation time. Especially when the size of the metamaterial beam is large, computational resource limitations may prevent the high-fidelity finite element method from obtaining a solution. However, after homogenization, the complex microstructural details no longer exist, thereby simplifying and accelerating the entire solving process.

By comparing the results obtained from the high-fidelity finite element method and the homogenization method with surface effect, it is demonstrated that the proposed homogenization method can effectively predict the deformation behavior of slender porous polymer beams subjected to bending loads. While ensuring accuracy, the online computation time is significantly reduced compared to the high-fidelity finite element method.

## 5. Conclusions

For porous polymer beams with complex microstructures, when the lattice size approaches the macroscopic thickness of the beam, the surface effect becomes significant and cannot be neglected in bending deformation. Moreover, the classical continuum assumption underlying conventional multiscale homogenization fails to hold in such beams, resulting in substantial relative errors in the predicted responses. In addition, the intricate microstructure of porous polymer beams leads to exorbitant computational costs when employing high-fidelity numerical methods. Therefore, a surface-driven homogenization method was developed to predict the bending response of these beams more accurately and efficiently. The key findings are summarized as follows:

(1) The mechanism of surface effect in thin porous polymer beams arises from the free surface regions at the top and bottom, where the lattice structures lack the interlocking interactions present in the core region subjected to bending loads, resulting in constraint deficiencies. Consequently, these surface regions exhibit weaker resistance to deformation. This effect becomes particularly significant when the macroscopic thickness of the metamaterial beam approaches the scale of the lattice constant, as the constraint deficiencies introduced by the free surfaces substantially reduce the beam’s ability to resist bending deformation.

(2) Based on the revealed mechanism, a homogenization model incorporating surface-driven moment of inertia was established. The magnitude of the surface effect was evaluated using two intrinsic parameters: the surface thickness *l* and the surface strength factor *k*. To calibrate these intrinsic parameters, a surface-driven homogenization method was developed based on the full-thickness representative volume element, which can predict the bending response of these beams more accurately and efficiently. Then, an online prediction framework based on the offline dataset generated by the surface-driven homogenization method was established for the size-dependent bending response.

(3) Numerical simulations using high-fidelity finite element analysis were conducted on porous polymer beams with varying thicknesses, porosities, and lattice constants. The results demonstrate that the predictions of beam deformation subjected to bending loads obtained by the homogenization method proposed in this work, which accounts for surface effect, align closely with those from the high-fidelity simulations. In contrast, the classical multiscale homogenization method exhibited a maximum prediction error of up to 1108%. Moreover, compared to the high-fidelity finite element method, the proposed homogenization approach not only significantly reduces computational time but also enables rapid analysis of complex metamaterial beam models.

In summary, this study not only elucidates the deformation mechanisms of porous polymer beams subjected to bending loads that are related to the surface-driven size effect, but also proposes an accurate and efficient tool to predict their bending behavior. These findings provide valuable guidance for the structural design and performance prediction of porous polymer beams in engineering applications.

## Figures and Tables

**Table 1:** Intrinsic parameters *k* and *l* of different lattices.

Types	*a* (mm)	$V_{p}$	$E^{b}$ (MPa)	k	l (mm)
Diamond	1	0.9	4.7	0.067	0.755
	1	0.8	53.3	0.103	0.585
	1	0.7	127.7	0.127	0.541
	1	0.6	234.2	0.146	0.512
Diamond	1	0.8	53.3	0.103	0.585
	2	0.8	53.3	0.103	1.170
	3	0.8	53.3	0.103	1.755
	4	0.8	53.3	0.103	2.340
Primitive	1	0.8	91.7	0.667	0.806
Fischer-Koch S	1	0.8	70.5	0.111	0.233
Split P	1	0.8	209.5	0.216	0.151

**Table 2:** Comparison of the computational accuracy and efficiency between the surface-driven homogenization method and high-fidelity finite element method.

Beam L×H	Beam 40 × 4			Beam 50 × 5			Beam 70 × 7		
	FEA	Homo	Difference (%)	FEA	Homo	Difference (%)	FEA	Homo	Difference (%)
Displacement (mm)	0.0375	0.0382	1.86	0.0266	0.0271	1.88	N/A	0.0169	-
Computation time (s)	2238	3	99.87	16,453	3	99.98	N/A	4	-

## Data Availability

The original contributions presented in this study are included in the article. Further inquiries can be directed to the corresponding authors.
